# Epitaxial Crystallization Behavior of Poly(butylene adipate) on Orientated Poly(butylene succinate) Substrate

**DOI:** 10.3390/polym10020110

**Published:** 2018-01-24

**Authors:** Haijun Wang, Zhijin Gao, Xi Yang, Kun Liu, Min Zhang, Xihuai Qiang, Xuechuan Wang

**Affiliations:** 1 College of Bioresources Chemical and Materials Engineering, Shaanxi University of Science and Technology, Xi’an 710021, China; my1063487993@163.com (Z.G.); yangxi5676@163.com (X.Y.); 18792615768@163.com (K.L.); qiangxh@sust.edu.cn (X.Q.); wangxc@sust.edu.cn (X.W.); 2 National Demonstration Center for Experimental Light Chemistry Engineering Education, Shaanxi University of Science and Technology, Xi’an 710021, China; 3 School of Environmental Science and Engineering, Shaanxi University of Science and Technology, Xi’an 710021, China; zhangmin@sust.edu.cn

**Keywords:** poly(butylene succinate), poly(butylene adipate), blend, epitaxy

## Abstract

The crystallization behavior of poly(butylene adipate) (PBA) in the sheared PBS/PBA blend, as well as on highly orientated poly(butylene succinate) (PBS) substrate, was studied by means of DSC, POM, Raman microscopy, and XRD. The results showed that the pre-existing orientated PBS crystals exhibit a very strong nucleation ability toward PBA as reflected by the increased crystallization temperature and the occurrence of heteroepitaxy and transcrystallization of PBA on the PBS substrate. The epitaxial crystallization of PBA on the PBS substrate results in the formation of α-form PBA crystals in any crystallization conditions.

## 1. Introduction

Epitaxial growth, as a specific case of surface-induced crystallization, originates from the interactions between two polymers at their interface. Polymer chains can only be orientionally deposited on the substrate along specific directions due to lattice matching [1]. It is well confirmed that epitaxy provides an effective way to control the crystal structure and orientation of a wide variety of crystallizable materials [2]. As an example, Lovinger has successfully controlled the crystallization of poly(vinylidene fluoride) from the melt into its piezoelectric β-form through epitaxy [3].

Aliphatic polyesters, exhibiting excellent biodegradability and biocompatibility, have been extensively used as environmental protection and biomedicine materials. Although the biodegradability of an aliphatic polyester originates from the chemical structure, its supermolecular structure, such as crystal form, lamellar thickness, and crystallinity, also affects its biodegradation rate and mechanical properties [4]. Therefore, it is important to control the supermolecular structure of aliphatic polyesters for the purpose of modulating their biodegradation rate. Epitaxial crystallization has been successfully applied to dominate the crystalline structure of aliphatic polyesters such as poly(lactic acid) (PLA), poly(ε-caprolactone) (PCL), and poly(ethylene adipate) (PEA) [5,6,7]. However, most of the substrates used (e.g., poly(ethylene) and isotactic polypropylene) are not biodegradable, so the composites of aliphatic polyesters with them are not fully biodegradable materials.

As a representative aliphatic polyester, poly(butylene adipate) (PBA) exhibits two kinds of crystalline modifications, denoted as α- and β-form. α-PBA has a monoclinic unit cell with dimensions of *a* = 6.70 Å, *b* = 8.00 Å, *c* (fiber axis) = 14.20 Å, and β = 45.5°. The β-PBA has an orthorhombic unit cell with dimensions of *a* = 5.05 Å, and *b* = 7.36 Å, *c* (fiber axis) = 14.67 Å [8]. Enzymatic degradation experiments have revealed that α-PBA degrades relatively faster than its β-form counterpart, which is probably attributed to the differences in the packing of chains, chain mobility, and the crystallization mechanism [9]. In addition, the ring-banded spherulites of PBA composed of (α + β) mixed crystals exhibit a slower degradation rate than β-PBA, suggesting that the biodegradability of PBA is also affected by the spherulitic morphology [10]. Therefore, it is necessary to control the crystal structure of PBA for the purpose of tailoring its biodegradation rate. In addition, the practical application of PBA has been limited because of its softness and slow crystallization rate [11]. It is of value to overcome the aforementioned problems in a useful and convenient manner.

Some studies on the polymorphic control of PBA crystallization have been reported. It is well known that the crystallization temperature is a key factor in determining the crystalline structure of PBA. The thermodynamically stable α-PBA crystallizes at a temperature above 31 °C and the metastable β-form crystallizes at a temperature below 29 °C. Upon annealing at elevated temperatures, the β-form might be able to transform into the α-form [12]. Adding nucleation agents can induce the formation of α- or β-form PBA and significantly accelerate the crystallization rate because of the reduced free energy required for nucleus formation [13,14]. In addition, blending PBA with other aliphatic polymers, such as poly(butylene succinate) (PBS) and poly(lactic acid) (PLA), is of scientific and industrial interest as a means of developing new biodegradable polymeric materials with desirable property combinations. The crystallization behaviors of PBA in its miscible blend with PBS and partially miscible blend with PLA have been investigated [15,16,17,18,19]. It was found that the miscibility significantly influences the morphology and crystalline structure of PBA.

As a model to study the crystallization behavior of PBA in its blends, more attention should be paid to the PBS/PBA blend. PBA is miscible with PBS in the melt state, so the final phase separation morphology of the PBS/PBA blend is induced by the crystallization of the two components. Because the melting temperature of PBS (114 °C) is far higher than that of PBA (54 °C), PBS always crystallizes before PBA. For the PBA component, the pre-existing PBS crystals greatly affect its spatial location. In our previous paper, the distribution of PBA within the PBS matrix was investigated in detail by atomic force microscopy [15]. It was found that PBA is located in the interlamellar region of PBS in the blend with PBA as a minority phase, but in the spherulitic or interfibrillar regions in PBS-rich blends. Yang et al. have investigated the effect of blend ratios on the crystallization PBA in PBS/PBA blends and found that the nonisothermal crystallization temperature of PBA is greatly reduced in PBS-rich blends, due to the spatial confinement of the pre-existing PBS crystals. This has been further proved by the morphological evidence. In another previous paper [16], in situ atomic force microscope (AFM) study of PBA lamellar growth showed that the growing PBA lamellae are often blocked by the PBS lamellae, so that they can only grow in many isolated interlamellar regions. However, Yang et al. have also found that in blends with a PBS content of less than 70%, the lowest crystallization temperature of the formation of α-form PBA decreases and the temperature range of the phase transition from the β- to α-PBA becomes wider [17]. It seems that PBS is favorable for the formation of α-form PBA. However, more evidence is needed to prove whether PBS crystals can induce the crystallization of PBA.

The well-established epitaxial growth of PBA has been found on non-biodegradable polyethylene [20], isotactic polypropylene [21], and Teflon substrates [22]. Until now, little research has been done on a biodegradable substrate. In this work, we have reported the epitaxial crystallization of PBA in its fully biodegradable blends with PBS, as well as on the surface of oriented PBS films. It was found that pre-existing orientated PBS crystals exhibit a very strong nucleation ability toward PBA and the epitaxial crystallization of PBA on the PBS substrate results in the formation of α-form PBA crystals under any crystallization conditions.

## 2. Experimental Section

### 2.1. Samples

Both PBS (*M*_w_ = 78,000 g/mol, *M*_n_ = 68,000 g/mol) and PBA (*M*_w_ = 12,000 g/mol) were purchased from the Sigma-Aldrich Company (Shanghai, China). The melting points of PBS and PBA were measured to be 114 and 54 °C. Blends of PBS and PBA were prepared by solution blending with chloroform as a common solvent. Both were dissolved in chloroform with desired mass proportions (total polymer concentration was 0.05 g/mL). The blend films were prepared by solution casting, producing films that were 1.5 μm in thickness. The average roughness of the blend films is 150 nm.

The blend samples were heated to and kept at 150 °C for 15 min to completely eliminate previous thermal history, then cooled to 80 °C for 3 h to ensure the complete crystallization of PBS, or sheared immediately with a silicon rubber plate at about 0.008 or 0.004 m/s. All the sheared and unsheared blend samples were finally cooled to 25, 27, 30, 35, and 40 °C for the crystallization of PBA.

### 2.2. Characterization

The morphologies of the specimens were mainly characterized via an Olympus BH-2 microscope (Olympus Optical Co., Tokyo, Japan) equipped with a Linkam LK-600 PM temperature controller. Differential scanning calorimetry (DSC) runs were performed on a Mettler DSC (Mettler Toledo, Giessen, Germany) under ultra pure nitrogen purge. Approximately 5 mg of samples was accurately weighed into an aluminium pan, the pans were hermetically sealed, and a pinhole was punched into the pan lid. Wide-angle X-ray diffraction (WAXD) analysis was carried out at room temperature using a Rigaku D/max 2500 VB2+/PC X-ray diffractometer (Rigaku, Tokyo, Japan) with Cu Kα radiation. Scanning was performed in reflection mode with 2θ from 10° to 40° at a rate of 1°/min with a step of 0.02°. In order to determine the detailed crystalline structure of samples, scanning microbeam 2D-WAXD measurements were performed for the sheared PBS film and PBA on the oriented PBS substrate sample. 2D-WAXD measurements were conducted at the beamline BL14B1 of the Shanghai Synchrotron Radiation Facility (SSRF, Shanghai, China). The X-ray beam with a wavelength of 0.1240 nm was used, and the distance from sample to detector was held at 172 mm. An X-ray CCD detector (Model Mar345) was employed to collect the 2D images. Detailed information about beamline BL14B1 can be found in Ref. [23]. Raman spectra were recorded using a Renishaw inVia Raman microscope (Renishaw, Gloucestershire, UK), equipped with a confocal microscope (Leica DM2500, Wetzlar, Germany). An Ar laser (wavelength 532 nm) was used for excitation. A 2400 lines per mm grating was utilized and the spectral resolution was 1 cm^−1^ during all measurements. The spatial resolution is 1 μm.

## 3. Results and Discussion

**Figure 1:**
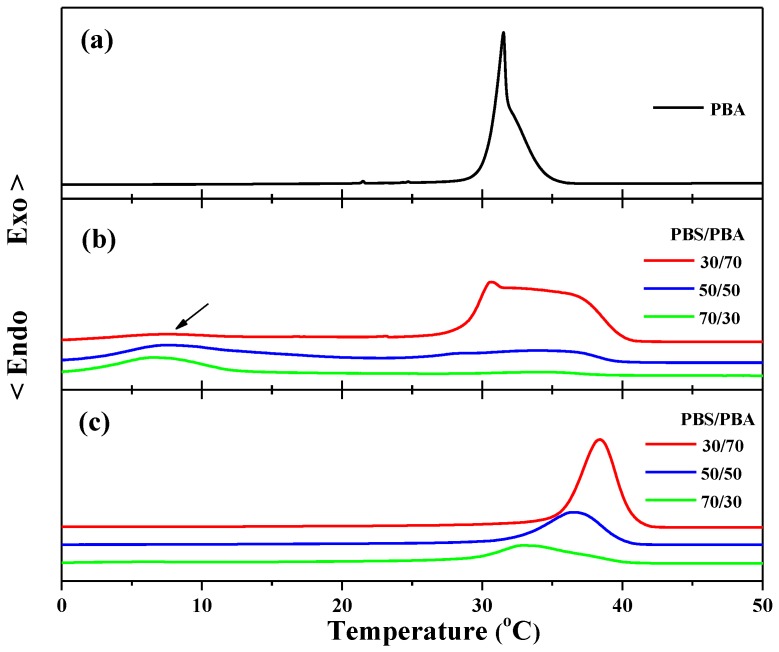
DSC cooling curves of (**a**) neat PBA, (**b**) PBS/PBA blends, and (**c**) sheared PBS/PBA blends from 80 °C at the cooling rate of 5 °C/min.

Figure 1a shows the nonisothermal melt crystallization of neat PBA at 5 °C/min. As seen in Figure 1a, neat PBA showed a two-stage crystallization behavior. The crystallization peak temperatures were 31.5 and 32.1 °C, corresponding to the α-form and β-form crystallization, respectively. Figure 1b shows the nonisothermal crystallization of PBA in PBS/PBA 30/70, 50/50, and 70/30 blends at 5 °C/min. As seen in Figure 1b, the fractional crystallization of PBA occurred in the PBS/PBA blend for all compositions. For the 30/70 blend, a very broad crystallization peak appeared at the temperature ranging from 27.5 °C to 40.0 °C and the onset crystallization temperature was about 5 °C higher than neat PBA. It was indicated that the pre-existing PBS crystals acted as the nucleation agent and promoted the crystallization of one part of PBA. However, there was another crystallization peak ranging from 0 °C to 9 °C, as indicated by the black arrow, and the onset crystallization temperature was about 20 °C lower than neat PBA, suggesting that the crystallization of this part of the PBA was confined by the pre-existing PBS crystals. The similar fractional and confined crystallization of PBA observed in the 30/70 blend also appeared in the 50/50 and 70/30 blends. In addition, it was also found that the relative intensity of the high crystallization peak to the low crystallization peak decreased with increasing PBS content from 30% to 70%, suggesting that the crystallization of PBA became more difficult in a PBS-rich blend. The confined crystallization of PBA should be ascribed to the spatial confinement within the PBS matrix. In our previous work, we have investigated in detail the morphological features of the PBS/PBA blends varying in blend ratio [15]. It was found that the PBA melt acts as a diluent, affecting the morphology of PBS, which in turn influences the phase separation behavior of PBA remarkably. For the PBA-rich blends, PBS only forms a spherulitic framework, filled in with the PBA lamellar crystals, indicating that interfibrillar mode is the main phase separation process. For the 50/50 blend, interlamellar and interfibrillar phase segregations take place simultaneously. For the PBS-rich blend, mainly interlamellar segregation of PBA occurs. That is, increasing the content of PBS results in the phase separation of PBA changing from interfibrillar to interlamellar mode. The crystallization capacity of PBA is explained by different phase segregation modes. Especially for those PBS/PBA blends with high PBS contents, the PBA component is dispersed in the isolated interlamellar domain, which makes its molecular chains difficult to diffuse from one domain to another. As a result, as shown in Figure 1b, the crystallization temperature of some PBA was greatly reduced, especially in PBS-rich blends.

The crystallization behavior of PBA, shown in Figure 1b, has clearly indicated that the crystallization behavior of PBA in its blend with PBS strongly depends on the compositions. Next, to study the effect of the oriented PBS component on the crystallization of PBA, their blends were cooled from 150 °C to 80 °C and then sheared with a silicon rubber plate. For the PBS component, shearing its supercooling melt led to the formation of oriented crystals. For the PBA component, the shear temperature was significantly above its melting point, so that shear stress applied to the molecular chains could be quickly relaxed. When the sheared blends were cooled below the melting temperature of PBA, the PBA component crystallized without the shear effect. Figure 1c shows the non-isothermal crystallization of PBA in the sheared 30/70, 50/50, and 70/30 PBS/PBA blends. By comparing Figure 1c with Figure 1b, we can find that the crystallization temperatures of PBA in the sheared PBS/PBA blends shifted to the higher temperature window for all blend compositions, and no fractional or confined crystallization was observed, reflecting that shear-induced oriented PBS crystals significantly promoted the crystallization of PBA.

**Figure 2:**
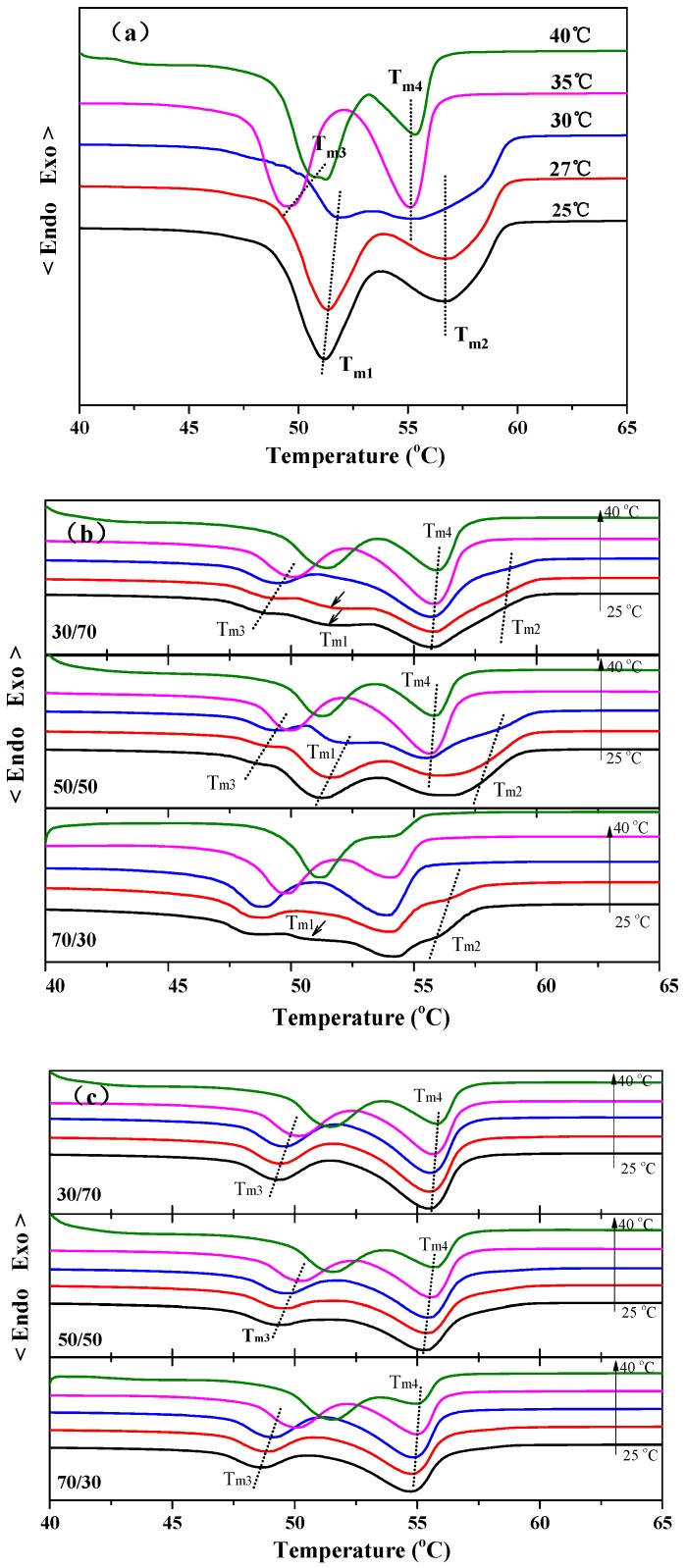
DSC melting curves of (**a**) neat PBA, (**b**) PBA in PBS/PBA blend, and (**c**) PBA in sheared blend after being melt-crystallized at various temperatures.

Figure 2a shows the DSC heating curves of neat PBA after being isothermally crystallized at different temperatures. As seen in Figure 2a, two melting peaks appeared at 51.2 °C (*T*_m1_) and 56.7 °C (*T*_m2_) at lower crystallization temperatures below 30 °C, while the other two melting peaks, marked as *T*_m3_ and *T*_m4_, appeared at 49.5 and 55.1 °C at higher crystallization temperatures above 30 °C. The DSC result shown in Figure 2a is similar to that of the previous reports [24]. According to the research of Gan et al., the multiple melting behavior of PBA varying with the crystallization temperatures corresponds to the β to α phase transformation. The two melting peaks marked as *T*_m1_ and *T*_m2_ can be attributed to the melt-recrystallization-remelt mechanism of β-form PBA crystals, while the peaks marked with *T*_m3_ and *T*_m4_ correspond to the melting of α-form PBA crystals [12].

Figure 2b shows the melting behavior of PBA in the PBS/PBA 30/70, 50/50, and 70/30 blends after being isothermally crystallized at different temperatures. As seen in Figure 2b, the characteristic melting peaks of α-type PBA crystals, i.e., *T*_m3_ and *T*_m4_, can be found at the temperatures ranging from 25 °C to 40 °C for all compositions, indicating that the pre-existing PBS crystals have a certain ability to induce the formation of α-form PBA crystals. However, two weak melting peaks of β-PBA crystals, i.e., *T*_m1_ and *T*_m2_, appeared at 25 and 27 °C for all compositions, suggesting that the crystal structure of PBA in its blends with PBS also rests on the crystallization temperatures.

The melting curves of PBA in the sheared PBS/PBA blend were shown in Figure 2c. It can be seen that the effect of crystallization temperatures and blend compositions on the melting behavior of PBA was greatly weakened. The PBA component in the sheared PBS/PBA blend only exhibits the characteristic melting peaks of α-form crystals for all set crystallization temperatures and blend compositions, suggesting that the oriented PBS component exhibits a very strong nucleation ability toward α-form PBA.

**Figure 3:**
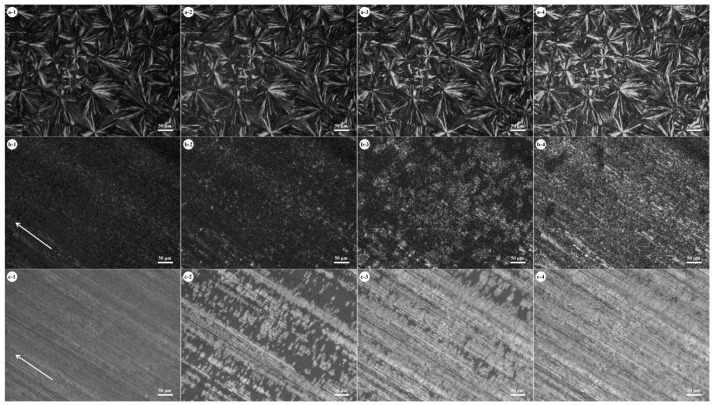
Crystallization of PBA in the 50/50 PBS/PBA blends at 40 °C. (**a-1**–**a-4**) unsheared PBS spherulites, (**b-1**–**b-4**) sheared PBS at 0.004 m/s, and (**c-1**–**c-4**) sheared PBS at 0.008 m/s. The sheared and crystallization temperature of PBS in all samples is 80 °C. The crystallization time of PBA: (−1) 0 min, (−2) 10 min, (−3) 20 min, and (−4) 40 min. The arrow in (**b-1**,**c-1**) indicates the shear orientation.

**Figure 4:**
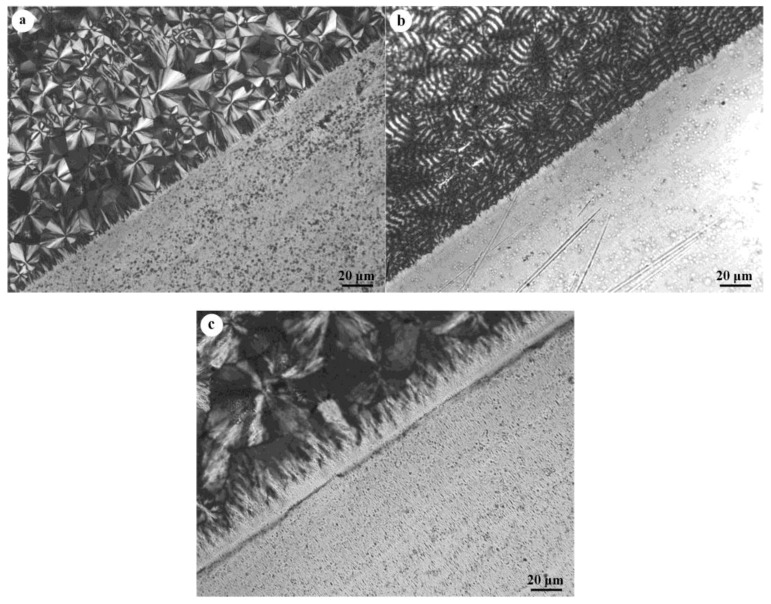
POM images of PBA crystallized at (**a**) 25 °C, (**b**) 30 °C, and (**c**) 40 °C on the oriented PBS substrates and glass substrates.

As seen in the above DSC results, shearing PBS/PBA blends led to an increased crystallization rate and preferential α-form PBA crystals. It seems that the shear-induced oriented PBS exhibits a very strong nucleation ability toward PBA. Shear-induced crystallization is of great technological importance in all fabrication processes such as injection moulding, extrusion, and film spinning. It has been found that the shear rate mainly affects the nucleation rate and speeds up the crystallization process [25]. Next, the effect of the shearing rate on the crystal structure of PBS and the crystallization process of PBA was investigated. Two shear rates, i.e., 0.004 and 0.008 m/s, were used. Figure 3 shows the crystallization process of PBA in PBS/PBA 50/50 blends after shearing PBS at different rates. Figure 3(a-1) shows the morphology of a PBS spherulite in an unsheared blend. As seen in Figure 3(a-1), PBS crystallized in compact spherulites and covered the whole area. The existence of a well-defined spherulite boundary indicates that no PBA melt was expelled into the interspherulitic regions. As shown in Figure 3(a-2–a-4), the overall intensity in birefringence of the PBS spherulites gradually increased with increasing crystallization time. This again demonstrates that the crystallization of PBA took place within the PBS spherulitic regions. Figure 3(b-1) shows the morphology of PBS which was sheared at 0.004 m/s and crystallized at 80 °C. As seen in Figure 3(b-1), the shish-kebab structure of PBS was formed after being sheared at 0.004 m/s, indicative of the increasing nucleation density. Moreover, the PBS crystals have a very low degree of orientation in most regions. Figure 3(b-2–b-4) shows the crystallization process of PBA. Through careful observation it can be found that PBA nucleated and crystallized on the PBS crystals. Accordingly, PBA also formed small crystals. Figure 3(c-1) shows the morphology of PBS, which was sheared at 0.008 m/s and crystallized at 80 °C. As seen in Figure 3(c-1), PBS still formed shish-kebab crystals under this crystallizaton condition. As seen in Figure 3(c-2–c-4), the PBA crystals grew in the direction perpendicular to the shearing orientation, suggesting that there is a relationship between the orientation directions of PBS and PBA crystals. In addition, the crystallization rate of PBA was also affected by the shearing rate. Comparing Figure 3(a-2) with Figure 3(b-2,c-2), one finds that the crystallization rate of PBA in the sheared blend at 0.008 m/s was faster. So, the effect of the shearing rate on the crystallization of PBS/PBA blends can be summarized. The shish-kebab structure of PBS was formed under shearing condition. With an increasing shearing rate, more PBS molecules were well aligned along the shearing direction and more highly oriented PBS crystals were preferentially formed. The highly oriented PBS crystals exhibited a strong nucleation ability and increased the crystallization rate of PBA.

Next, to elucidate the heterogeneous nucleation of PBA on oriented PBS crystals, the crystallization of PBA on the surface of a highly oriented PBS thin film was investigated from the perspective of epitaxial crystallization. The crystallization morphologies and crystal structure of PBA on a highly oriented PBS substrate were investigated by polarized light microscope (POM) and WAXD. Figure 4a–c presents the POM micrographs of PBA crystallized at 25, 30, and 40 °C, respectively. For a direct comparison, a boundary region was chosen with the thin PBS film located at the lower right corner. From these POM pictures, it can be clearly seen that the crystallization of PBA on a highly oriented PBS substrate yielded a quite different supermolecular structure as compared with a glass substrate. PBA crystallized on glass produced spherulites, the size of which increased with the crystallization temperature. However, on the PBS substrate, the nucleation density was so large that no individual PBA spherulites can be identified for all set crystallization temperatures. Additionally, the transcrystalline layers of PBA induced by PBS can be clearly seen on the boundary. These implied that the PBS substrate exhibits an active nucleation ability toward PBA.

**Figure 5:**
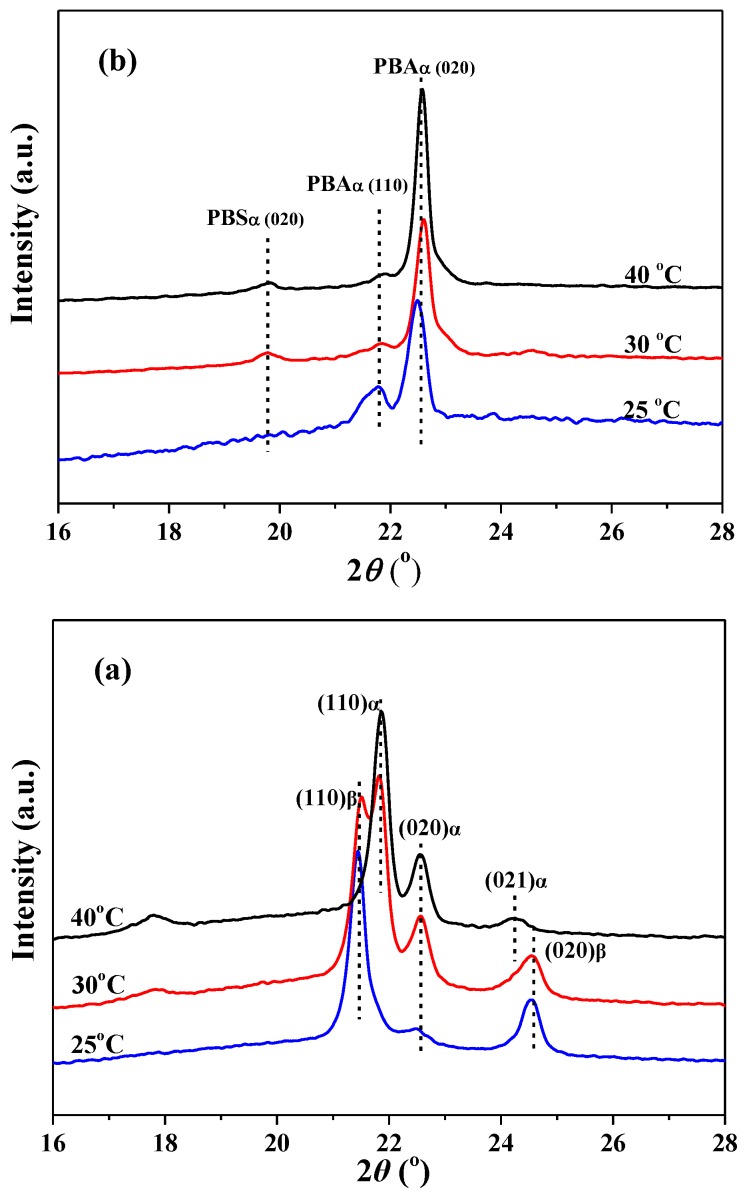
WAXD images of PBA crystallized on (**a**) glass and (**b**) PBS substrates at different temperatures.

Figure 5 presents the X-ray diffraction measurement of PBA crystallized on glass and PBS substrates. As seen from Figure 5a, the WAXD of PBA crystallized on the glass substrate shows reflection peaks at 2θ of 21.9° [α(110)], 22.5° [α(020)], and 24.2° [α(021)] at 40 °C, demonstrating the formation of α-form crystals at a high temperature [12]. The WAXD of PBA crystallized on the glass substrate at 25 °C shows the reflection peaks at 2θ of 21.4° [β(110)] and 24.5° [β(020)], indicating the crystallization of PBA in β-form [12]. The WAXD of PBA crystallized at 30 °C displays the reflections of both α- and β-form PBA crystals, implying the coexistence of two types of crystals. Figure 5b shows the WAXD of PBA crystallized on a highly oriented PBS substrate at different temperatures. As reported, neat PBS has two main characteristic diffraction peaks, at 2θ of 19.8° and 21.4°, corresponding to the (020) and (021) planes, respectively [26]. However, the intensity of the two typical diffraction peaks of PBS was greatly weakened because the PBS substrate film was very thin and its surface was almost completely covered by a thicker PBA film. The WAXD of PBA crystallized on a PBS substrate only shows the reflection peaks of α-form crystals at all set crystallization temperatures, suggesting that the epitaxial crystallization of PBA on oriented PBS substrate results in the formation of α-form PBA crystals, regardless of the crystallization temperature. By comparing Figure 5a with Figure 5b, it was found that the α(020) reflection is strengthened, while the α(110) reflection is weakened remarkably, a behavior which is probably related to the preferred orientation of PBA on the PBS substrate.

**Figure 6:**
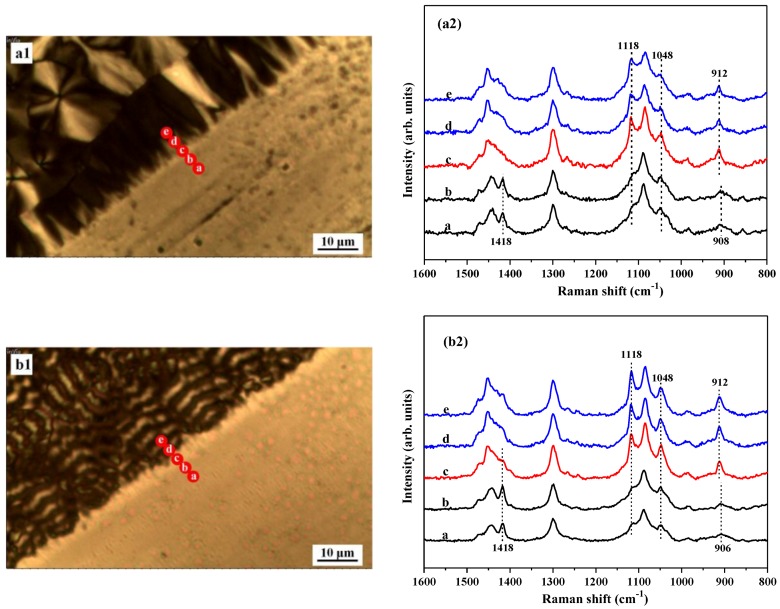
Raman spectra of PBA crystallized at (**a1**,**a2**) 25 °C and (**b1**,**b2**) 30 °C in the interfacial region from PBS to glass substrate.

To clarify the structural evolution in the interfacial region from PBS to glass substrate, Raman spectroscopy was used. For this purpose, the Raman band assignments of PBA in its different phases were adopted. It was reported that the α-form PBA has the characteristic FTIR bands at 909, 1170, 1260, 1399, 1369, 1419, 1462, and 1731 cm^−1^. Additionally, β-form PBA shows characteristic bands at 910, 930, 960, 1263, 1370, 1401, 1417, 1464, and 1729 cm^−1^ [27]. According to these assignments, the crystalline structure of PBA can be easily classified. Figure 6(a1,b1) is the mapping images of the PBA samples crystallized at 25 and 30 °C. As marked with red dots in Figure 6(a1,b1), the Raman signals were collected across the interface region from PBS to glass substrates. Figure 6(a2,b2) shows the corresponding Raman spectra of the samples. As seen in Figure 6(a2), on glass substrate, typical Raman bands of β-form PBA at 1118, 1048, and 912 cm^−1^ were clearly observed, indicating that PBA forms β-form crystals on glass substrate at 25 °C. On the boundary between glass and PBS substrate, it was found that the characteristic Raman bands of β-PBA at 1118, 1048, and 912 cm^−1^ are gradually weakened, but the characteristic Raman bands of α-PBA at 1418 and 908 cm^−1^ were strengthened. On the PBS substrate, only the characteristic Raman bands of α-PBA at 1418 and 908 cm^−1^ were observed, suggesting that α-PBA crystals were formed on the PBS substrate. As shown in Figure 6(b1,b2), a similar phase transformation from β-crystals on glass to α-crystals on PBS substrate were also found at 30 °C.

**Figure 7:**
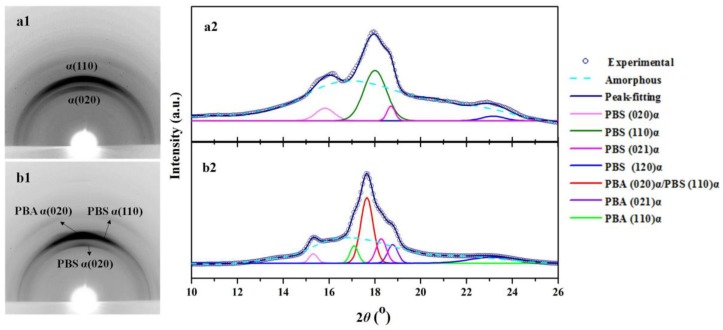
Typical 2D WAXD patterns of (**a1**) oriented PBS film, (**b1**) PBA crystallized at 25 °C on the oriented PBS substrates, and their corresponding intensity profiles of (**a2**,**b2**).

For the 2D WAXD patterns shown in Figure 7, the incident X-ray beam was perpendicular to the direction of oriented PBS substrates, and hko-arcs are observed along the meridian in the 2D WAXD patterns instead of isotropic diffraction rings, implying orientation of the samples. In the oriented PBS 2D WAXD pattern (Figure 7(a1)), the arcs indexed as (020)_α-PBS_ and (110)_α-PBS_ diffractions were detected, indicating the high orientation of the melt-sheared PBS film. As shown in Figure 7(b1), (020)_α-PBA_ and (110)_α-PBS_ diffraction arcs overlapped, suggesting that the *b* axis of the PBA may be in contact with the PBS substrate. The α-form PBA has a monoclinic unit cell with dimensions of *a* = 6.70 Å, *b* = 8.00 Å, *c* (fiber axis) = 14.20 Å, and β = 45.5°. The α-form PBS also has a monoclinic unit cell with dimensions of *a* = 5.23 Å, *b* = 9.12 Å, *c* (chain axis) = 10.90 Å, and β = 123.9° [8]. Comparing the crystal structure of α-PBS (Figure 7(a2)) and α-PBA (Figure 7(b2)), matching between the interplane distances of (020)_α-PBA_ and (110)_α-PBS_ with a mismatching of 2.0%, which is well within the upper limit (~15%) for polymer epitaxy, can be found. This indicates that lattice matching may also play an important role in the epitaxial crystallization of PBA on PBS substrate.

## 4. Conclusions

The crystallization behaviors of poly(butylene adipate) (PBA) in unsheared and sheared PBS/PBA blends, as well as on highly orientated PBS substrates, were studied by means of DSC, POM, Raman microscopy, and XRD. The results show that the fractional and confined crystallization behavior of PBA in its blend with PBS greatly depends on the blend composition. In the unsheared PBS/PBA blend, a large amount of pre-existing PBS crystals impede the crystallization of PBA, but a minority of PBS crystals can act as the nucleation agent and promote the crystallization of PBA. In the sheared PBS/PBA blend, the oriented PBS crystals exhibit a very strong nucleation ability toward PBA, as reflected by the increased crystallization temperature, elimination of fractional crystallization, and the occurrence of heteroepitaxy and transcrystallization of PBA on the PBS substrate. The epitaxial crystallization of PBA on PBS substrate results in the formation of α-form PBA crystals under any crystallization conditions. This provides a new method for manipulating the crystalline structure and orientation of PBA in its fully biodegradable blend with PBS.
